# Field Testing Integrated Interventions for Schistosomiasis Elimination in the People's Republic of China: Outcomes of a Multifactorial Cluster-Randomized Controlled Trial

**DOI:** 10.3389/fimmu.2019.00645

**Published:** 2019-04-03

**Authors:** Gail M. Williams, Yue-Sheng Li, Darren J. Gray, Zheng-Yuan Zhao, Donald A. Harn, Lisa M. Shollenberger, Sheng-Ming Li, Xinglin Yu, Zeng Feng, Jia-Gang Guo, Jie Zhou, Yu-Lan Dong, Yuan Li, Biao Guo, Patrick Driguez, Marina Harvie, Hong You, Allen G. Ross, Donald P. McManus

**Affiliations:** ^1^School of Public Health, University of Queensland, Brisbane, QLD, Australia; ^2^Molecular Parasitology Laboratory, Infectious Diseases Division, QIMR Berghofer Medical Research Institute, Brisbane, QLD, Australia; ^3^World Health Organisation Collaborating Centre for Research and Control of Schistosomiasis in Lake Region, Hunan Institute of Parasitic Diseases, Yueyang, China; ^4^Research School of Population Health, Australian National University, Canberra, ACT, Australia; ^5^Department of Infectious Diseases, College of Veterinary Medicine and Center for Tropical and Emerging Global Diseases, University of Georgia, Athens, GA, United States; ^6^Department of Biological Sciences, Old Dominion University, Norfolk, VA, United States; ^7^Chinese Centre for Disease Control and Prevention, National Institute of Parasitic Diseases, Shanghai, China; ^8^Centre of Cell and Molecular Biology Experiment, Xiangya School of Medicine, Central South University, Changsha, China; ^9^Menzies Health Institute, Griffith University, Gold Coast, QLD, Australia; ^10^International Centre for Diarrhoeal Disease Research (ICDDR), Dhaka, Bangladesh

**Keywords:** schistosomiasis japonica, China, CRT, bovine vaccine, mathematical modeling

## Abstract

Despite significant progress, China faces the challenge of re-emerging schistosomiasis transmission in currently controlled areas due, in part, to the presence of a range of animal reservoirs, notably water buffalo and cattle, which can harbor *Schistosoma japonicum* infections. Environmental, ecological and social-demographic changes in China, shown to affect the distribution of oncomelanid snails, can also impact future schistosomiasis transmission. In light of their importance in the *S. japonicum*, lifecycle, vaccination has been proposed as a means to reduce the excretion of egg from cattle and buffalo, thereby interrupting transmission from these reservoir hosts to snails. A DNA-based vaccine (SjCTPI) our team developed showed encouraging efficacy against *S. japonicum* in Chinese water buffaloes. Here we report the results of a double-blind cluster randomized trial aimed at determining the impact of a combination of the SjCTPI bovine vaccine (given as a prime-boost regimen), human mass chemotherapy and snail control on the transmission of *S. japonicum* in 12 selected administrative villages around the Dongting Lake in Hunan province. The trial confirmed human praziquantel treatment is an effective intervention at the population level. Further, mollusciciding had an indirect ~50% efficacy in reducing human infection rates. Serology showed that the SjCTPI vaccine produced an effective antibody response in vaccinated bovines, resulting in a negative correlation with bovine egg counts observed at all post-vaccination time points. Despite these encouraging outcomes, the effect of the vaccine in preventing human infection was inconclusive. This was likely due to activities undertaken by the China National Schistosomiasis Control Program, notably the treatment, sacrifice or removal of bovines from trial villages, over which we had no control; as a result, the trial design was compromised, reducing power and contaminating outcome measures. This highlights the difficulties in undertaking field trials of this nature and magnitude, particularly over a long period, and emphasizes the importance of mathematical modeling in predicting the potential impact of control intervention measures. A transmission blocking vaccine targeting bovines for the prevention of *S. japonicum* with the required protective efficacy would be invaluable in tandem with other preventive intervention measures if the goal of eliminating schistosomiasis from China is to become a reality.

## Introduction

Schistosomiasis, caused by *Schistosoma japonicum*, threatened many millions of people in the People's Republic of China (P.R. China) prior to initiation of the national schistosomiasis control program in the 1950s when nation-wide surveys identified 12 provinces as endemic, mostly in the south, with 12 million people infected (600,000 advanced cases), and 100 million people at risk of infection (1, 2). Based on the habitat of the *Oncomelania hupensis* snail intermediate hosts, the endemic areas for zoonotic schistosomiasis japonica in the P.R. China are classified as one of three area types: the lakes and marshlands, situated in Hunan, Jiangxi, Anhui, Jiangsu, and Hubei (currently accounting for over 95% of the snail habitats in the country); hilly and mountainous regions of the upper reaches of the Yangtze River in Sichuan and Yunnan (4.91%); and the plains region, with waterway networks (0.03%), mainly located along the Yangtze River (2). The majority of *S. japonicum* transmission, which occurs annually from April to October/early November, is predominantly around the Dongting Lake (Hunan Province) and Poyang Lake (Jiangxi Province), China's two largest lakes (2).

The national schistosomiasis control program for the P.R. China achieved transmission control by the mid-1980s, introduced mass chemotherapy and morbidity control by the early 2000s, and has now transitioned to integrated control approach (3). The Chinese government included schistosomiasis, together with three other diseases (AIDS, TB, and Hepatitis B), in the 11th and 12th 5 Year Plan (2005–2015) as one of four main infectious diseases to target for elimination (4). As a result, control efforts were intensified at this time with the aim of reducing the overall infection prevalence to 1% by 2015. The control/elimination program employed a multi-sectoral, collaborative approach that embraced environmental modification, snail control, health education, and chemotherapy. Over the next 10 years, five provinces achieved the national transmission interruption target (i.e., zero infections in humans, animals and snails), with seven other provinces achieving the national transmission control target (i.e., infection prevalence of <1%). Areas endemic for schistosomiasis were reduced from 12 provinces (Jiangsu, Zhejiang, Anhui, Jiangxi, Fujian, Hunan, Hubei, Guangdong, Sichuan, Yunnan, Shanghai, Guangxi) to seven provinces (Hubei, Hunan, Anhui, Jiangxi, Jiangsu, Sichuan, Yunnan) (5–8). Schistosomiasis cases declined substantially from 240,000 (30,000 advanced cases) by the end of 2012, and to 77,190 in 2015; no acute cases have been reported since 2015 (9, 10).

Despite these great strides and considerable achievements, China still faces the challenge of re-emerging transmission in currently controlled areas due, in part, to the presence of more than 40 animal reservoir species capable of harboring *S. japonicum* infections (2, 3), with over 75% of schistosomiasis transmission attributed to water buffalo and cattle. Recent environmental, ecological, and social-demographic changes such as the effects of global warming and the construction of the Three Gorges Dam on the Yangtze, recently shown to affect the distribution of oncomelanid snails, also have the potential to impact on transmission (11, 12).

In addition to the employment of the customary interventions (chemotherapy, mollusciciding, health education), the current integrated control strategy focuses on interrupting bovine-snail transmission by replacing bovines with mechanized farming, prohibiting the pasturing of animals near lakes and rivers, raising livestock in herds, and creating safe grazing areas. Supplying safe water, building lavatories and latrines, constructing marsh-gas pools, and providing fecal-matter containers for fishermen's boats are aimed at interrupting human-snail transmission (13). However, some interventions may not be effective for all areas. For example, replacing bovines with tractors may not be practical for particular terrains (14).

In light of their importance as major reservoirs for *S. japonicum*, vaccination of bovines has been proposed as a tool to assist in long-term prevention (2, 15, 16), supported by mathematical modeling (17); the intervention would be particularly applicable for areas where mechanical farming is unsuitable. Vaccination can reduce egg excretion from cattle and buffalo, thereby interrupting transmission from bovines to snails. A schistosome plasmid DNA vaccine (SjCTPI-Hsp70) our team developed showed very good efficacy against *S. japonicum* in Chinese water buffaloes, when it was co-administered with an IL-12 expressing plasmid as adjuvant (18). The present paper reports the results of a double-blind cluster-randomized controlled trial (CRT) using a multi-factorial randomized design around the Dongting Lake area of Hunan province in P.R. China over the period 2010–2014. The trial aimed to determine the impact of a combination of human mass chemotherapy, snail control through mollusciciding and the SjCTPI bovine vaccine, on the transmission of *S. japonicum;* the trial profile (Supplementary Figure 1) and baseline results were reported previously (19). The design of the trial (Table 1) is technically known as a “split-plot” design. Developed by RA Fisher in 1925 (20), it was initially used for agricultural testing of fertilizers—where one set of treatments is allocated randomly to predefined “plots” of land (usually predefined subdivisions of a larger area, e.g., gridded squares), then these plots are each subdivided to sub-plots which are then used to test another set of treatments. As far as we are aware this type of factorial design has not been applied hitherto in field/clinical trials of populations before. Furthermore, this study is the first to report on the outcomes of a CRT to test a schistosomiasis transmission blocking vaccine in the field.

**Table 1:** Intervention matrix conceptually highlighting the factorial study design used in village groups A–F. Reprinted from Gray et al. (19) with permission from Elsevier.

**Intervention**	**Bovines given active vaccine**	**Bovines given placebo vaccine**
Mollusciciding	2 villages (A)	2 villages (B)
Human treatment	2 villages (C)	2 villages (D)
Neither	2 villages (E)	2 villages (F) control

“*A” villages received mollusciciding + bovine Schistosoma japonicum triosephosphate isomerase (SjCTPI) vaccine. “B” villages received mollusciciding + bovine placebo vaccine. “C” villages received human treatment (praziquantel, PZQ) + bovine SjCTPI vaccine. “D” villages received human treatment (PZQ) + bovine placebo vaccine. “E” villages received bovine SjCTPI vaccine. “F” villages received bovine placebo vaccine*.

## Materials and Methods

### Trial Setting, and Baseline and Follow-Up Surveys

The CRT was conducted in 12 administrative villages (Baitang, Ganzhou, Nandi, Shuangzhou, Yuantan, Dongguo, Tuandong, Changjiang, Tuqiao, Xihuyuchang, Chunfeng, and Beihu) in the Dongting Lake area in Hunan province from 2010 to 2014, and aimed to quantify the effects of integrated interventions for eliminating *S. japonicum* (19). Hunan was selected as the study site because it is an endemic province, with relatively high infection rates, comparatively, in a setting of pre-elimination, many counties in the province are located close to the Lake, and a large stable rural population is at risk of being infected or re-infected with *S. japonicum*. A baseline survey was conducted in October to November 2010 and the interventions were implemented in six intervention groups from 2011 to 2013. In brief, the baseline survey included: (i) Collection of a human stool sample, which was tested for *S. japonicum* infection using the miracidial hatching test (MHT) (two stools/three hatches per stool-30 g feces/hatching; read blind) (19), after which the Kato Katz thick smear technique was used on positive samples to determine the infection intensity [Geometric Mean Eggs per Gram (GMEPG)] (19); (ii) A questionnaire survey of village residents to collect information on demographic variables, medical history and water contact (19); (iii) Collection of bovine stool samples, which were tested for *S. japonicum* infection using the MHT (one stool/three hatches −50 g feces/hatching; read blind), followed by the formalin-ethyl acetate sedimentation-digestion (FEA-SD) test for positive samples to determine infection intensity (19); and (iv) Oncomelanid snail surveys using the Chinese method of random quadrat sampling applied to marshland areas for each village (19).

Villages were then pair-matched for the CRT based on historical prevalence and transmission ecology (19). One of three intervention types (no specific intervention (control), human mass praziquantel (PZQ) treatment, mollusciciding) was randomly assigned to each pair to achieve two pairs per intervention type. Within each pair, one village was randomly assigned the active vaccine for vaccinating bovines and the other village received a placebo vaccine. Bovines received the priming SjCTPI DNA vaccine and protein boost, or placebo control, in 2011 with subsequent booster vaccinations or placebo controls given in 2012 and 2013. All animals present on the marshland at any particular time with active water contact received the vaccine or placebo. Full details of the production and formulation of the SjCTPI vaccines (plasmids encoding SjCTPI-HSP70 and UMVC3-mIL12 and recombinant SjCTPI) and placebo control vaccine, the prime (DNA) and boost (recombinant protein) vaccination regimen, and the procedures for injecting bovines with the vaccine/placebo formulations have been provided (19).

The investigators, including the research team, and study participants were blind to the vaccine allocation. Following the baseline survey, all residents (40 mg/kg) and bovines (25 mg/kg) were treated with PZQ. Human mass treatment with PZQ was carried out annually in the two randomly selected village pairs. Mollusciciding (following the annual snail surveys in March/May, 2011, 2012, 2013, and 2014 in two other randomly chosen village pairs) targeted snail “hotspots” (areas close to human habitation with maximum or daily access to both human and bovines in an environment favored by snails), which were sprayed with niclosamide (2 g/m^2^) annually.

### Study Subjects, Data Collection, and Management

**Figure 1:**
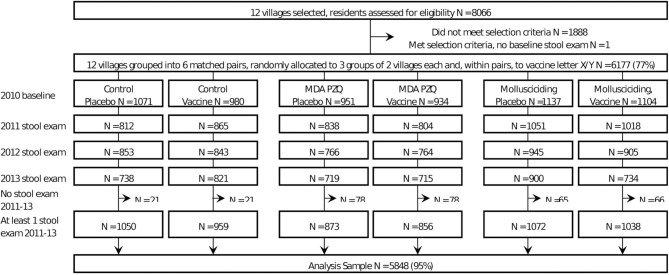
Trial recruitment, intervention allocation, and follow-up.

Questionnaire survey data and results of the stool examinations were collected during the baseline survey in October-November 2010 and at follow-ups in October-December from 2011 to 2013. Baseline and follow-up data for each village were cleaned and combined for the analyses in this paper. Based on specified inclusion criteria (19), 6,177 participants (out of a total of 8,066 villagers in the 12 villages assessed for eligibility at baseline) were selected to participate in the CRT (Figure 1). Inclusion criteria for each subject included: (i) age 5–65 years; (ii) had been a resident in the village for >12 months; (iii) would not be migrating in the next 4 years; (iv) continuously resident in the study area over the study period; (v) the resident provided informed consent; and (vi) minors had the informed consent of their parent/guardian.

### Bovine Serology

The majority of bovines involved in the trial were water buffaloes with only a small proportion being cattle. Sentinel water buffaloes, selected randomly from all villages (total = 300 animals), were tagged and bled periodically. Blood samples were taken prior to the commencement of vaccination (January, 2011); after the priming vaccination and primary boost (May, 2011), and then after post-boosts in May 2012 and April 2013. Serum was harvested from each blood sample and stored at −80°C for serology which was performed at trial completion. Western blotting (on a subset of sera) and indirect IgG ELISA were used to determine levels of specific anti-SjCTPI antibodies and anti-*S. japonicum* SEA (soluble egg antigens, SjCSEA) antibodies in the bovine sera over the course of the trial (18, 19). In brief, 96-well ELISA plates (Maxisorp Immuno; ThermoFisher, Scoresby, Australia) were coated with 10 μg/ml of recombinant SjCTPI (18) or SjCSEA (21) diluted in carbonate coating buffer. The plates were incubated at 37°C for 1 h and then washed three times with 0.05% Tween20 in PBS (PBST). The plates were blocked with 2% casein in coating buffer for 1 h at 37°C and then washed. The freshly thawed bovine serum was diluted 1:100 in 1% milk in PBST and duplicates were incubated for 1.5 h at 37°C. The plates were then washed four times including two times with 5 min of soaking. Diluted detection antibody (1:10,000 rabbit anti-bovine IgG-HRP A5295, Sigma-Aldrich, Castle Hill, Australia) was added to individual wells and the plates incubated for 1 h at 37°C. The plates were washed four times with PBST. The reaction was detected with TMB (3,3′5,5′-tetramethylbenzidine) substrate (Scientific Research Special SRS, Changsha, China) and stopped after 10 min at room temperature with 5% (v/v) hydrochloric acid. Positive controls were included on each SjCTPI and SjCSEA ELISA plate. The positive control wells for the SjCTPI plates were probed with a biotin-conjugated anti-His antibody (mouse IgG2a, clone HIS-1 H1029, Sigma-Aldrich; targeting the His-tag on the recombinant SjCTPI protein) followed by a streptavidin-HRP (Pharmingen, San Diego, USA) detection step. The positive control wells for the SjCSEA plates were probed with a pool of highly reactive sera collected from bovines from schistosomiasis japonica-endemic areas and detected with the same method used for the other serum samples. The negative control wells for both ELISA assays comprised a pool of sera obtained from bovines collected from areas non-endemic for schistosomiasis japonica. The plates were read with a plate reader at OD_450_ and the duplicates averaged. To account for inter-plate variation, the OD_450_ of the samples were normalized by multiplying the ratio of the average of the positive controls across all the plates to the plate-specific positive controls. Anti-SjCTPI and anti-SjC SEA antibody OD_450_ levels for the control samples (non-endemic, endemic positive and blank wells) are shown in Supplementary Figures 2, 3.

### Snail Surveys

Snail surveys used the Chinese method of random quadrat sampling (0.11 m^2^ sized frames, 20 m between frames) located on the marshland areas appropriate for each village (19).

### Statistical Methods

The study was designed to have 80% power to detect the intervention effect, using intervention efficacy estimations for vaccine (50%), human chemotherapy (85%), and mollusciciding (75%). Calculations assumed an infection rate of 5–10%, and a design effect of 1.5 to account for cluster effects, and 10% loss-to-follow-up. A SAS program was written to carry out the randomization to each intervention group. Analyses of human infection were restricted to those who satisfied the initial inclusion criteria and had baseline questionnaire and stool results and at least one follow-up stool result.

For intervention assessment the primary outcome was human *S. japonicum* infection status at follow-ups in 2011, 2012, and 2013, with a positive infection defined as the presence of at least one miracidium from the MHT (19). This was analyzed using a logistic regression model to compare interventions (grouped as control arm, human chemotherapy arm, mollusciciding arm, and vaccine group) over time, using a year-intervention group interaction. The models used Generalized Estimating Equations (GEEs) to take account of clustering and repeated measures, with an unstructured correlation structure for the latter. The model included age group, sex, and baseline infection as covariates. Contrasts were constructed to estimate the overall effect of each intervention (averaged over other intervention groups) for each follow-up year, and overall years. Subgroup effects were similarly estimated for the effect of each intervention within each other intervention group. Odds ratios and 95% confidence intervals were estimated. Sensitivity analyses were conducted by restricting analyses based on water contact exposure (at least monthly exposure), occupation (farming and/or fishing) and season of exposure (summer). Because of the large amount of missing data on water contact, an imputed water contact was calculated, based on the “last-observation-carried-forward” method.

All data management and analyses used SAS (r) Proprietary Software 9.4 (TS1M2) [Copyright (c) 2002–2012 by SAS Institute Inc., Cary, NC, USA, Licensed to UNIVERSITY OF QUEENSLAND—EAS, Site 10005036]. Data were double entered into a specially designed Microsoft Access-based database we developed (19); electronic copies of all entered data were saved offline and backup paper duplicates were stored in a secure location.

### Mathematical Modeling

The modeling of schistosomiasis has important implications when considering different control options. Models can predict disease spread, the usefulness of different strategies for treatment coverage, the effect of vaccines and the costs of control. The elimination of schistosomiasis in China will rely on a combination of different integrated control options, such as mollusciciding, environmental modification, drug treatment regimens, health education, improved sanitation, and bovine vaccination could provide an important tool to assist in long term prevention. That such a strategy could prove realistic gains support from studies in China that show that the human-snail-human cycle of transmission is less prominent than the animal-snail-human cycle in sustaining schistosome infection (16). A mathematical model was used to predict longer term effects post-trial. This model is based on that developed by Williams et al. (17), with extensions to include births and deaths of all hosts, with constant net population, and an additional compartment for all hosts to represent infection prior to infectiousness (22). All models included a single human and bovine treatment with efficacy 85% at the start of the intervention period (baseline). The effects of no further intervention and three annual cycles of human mass drug administration (HMA, coverage 80%) and mollusciciding (efficacy 80%, coverage 90%) were modeled separately. The combination of human MDA and mollusciciding, not one of the trial interventions, was also modeled. For each of these scenarios, vaccine effects were modeled with 0, 25, 50, and 75% efficacy and 80% coverage.

## Results

### Humans

A total of 8,066 residents from the 12 selected villages were screened, with 6,177 satisfying the selection criteria (Figure 1). Those for whom a stool sample was tested at each follow-up year are shown in Figure 1. The number of follow-ups per person varied from one to three, with some being available for a later follow-up, but not an earlier one. The final analysis set, restricted to those with at least one follow-up stool result, comprised 5,848 residents (Figure 1).

The overall prevalence of infection was 6.5% with infection intensity in those infected being 15.5 GMEPG at baseline. Baseline prevalence and infection intensity in 2010 and cumulative prevalence and infection intensity in 2011–2013 are shown by intervention arm (and vaccine group) in Tables 2, 3. Human infection prevalence did not vary significantly among intervention arms at baseline and prevalence decreased over time in all groups, most noticeably in 2012 and 2013.

**Table 2:** Human infection (%) by intervention group and year.

**Group**	**2010**		**2011**		**2012**		**2013**	
	**Number tested**	**Prevalence (95% CI)**	**Number tested**	**Prevalence (95% CI)**	**Number tested**	**Prevalence (95% CI)**	**Number tested**	**Prevalence (95% CI)**
^*^Control	2,009	6.7 (5.7, 7.8)	1,677	4.8 (3.5, 6.6)	1,696	3.5 (2.3, 5.1)	1,559	2.5 (1.7, 3.6)
Placebo vaccine	1,050	6.6 (5.2, 8.2)	812	3.3 (2.2, 4.9)	853	2.1 (1.3, 3.4)	738	1.8 (1.0, 3.0)
Active vaccine	959	6.8 (5.3, 8.6)	865	6.5 (4.9, 8.7)	843	5.2 (3.7, 7.1)	821	3.3 (2.3, 4.8)
**Human treatment**	1,729	6.5 (5.5, 7.8)	1,642	5.5 (4.0, 7.4)	1,530	2.5 (1.6, 3.8)	1,434	1.6 (1.0, 2.5)
Placebo vaccine	873	5.0 (3.8, 6.7)	838	4.8 (3.4, 6.6)	766	2.7 (1.7, 4.2)	719	1.9 (1.2, 3.3)
Active vaccine	856	8.1 (6.4,10.1) 10.1)	804	6.4 (4.7, 8.6)	764	2.3 (1.4, 3.8)	715	1.3 (0.7, 2.4)
**Mollusciciding**	2,110	6.2 (5.3, 7.3)	2,069	5.7 (4.3, 7.6)	1,850	2.8 (1.9, 4.2)	1,634	1.5 (1.0, 2.4)
Placebo vaccine	1,072	6.5 (5.2, 8.2)	1,051	6.5 (4.9, 8.4)	945	3.5 (2.4, 5.0)	900	1.9 (1.2, 3.0)
Active vaccine	1,038	5.9 (4.6, 7.5)	1,018	5.2 (3.8, 6.9)	905	2.3 (1.5, 3.6)	734	1.2 (0.6, 2.3)
Total	5,848	6.5 (5.9, 7.1)	5,388	5.3 (4.5, 6.4)	5,076	2.9 (2.3, 3.7)	4,637	1.8 (1.3, 2.4)

* *Control, group received the placebo or SjCTPI vaccine only*.

**Table 3:** Human infection intensity in infected persons (Geometric Mean EPG; GMEPG) by intervention group and year.

**Group**	**2010**		**2011**		**2012**		**2013**	
	**Number positive**	**GMEPG** **(95% CI)**	**Number** **positive**	**GMEPG** **(95% CI)**	**Number** **positive**	**GMEPG** **(95% CI)**	**Number** **positive**	**GMEPG** **(95% CI)**
^*^Control	134	19.7 (13.7, 28.4)	84	32.1 (21.4, 48.1)	53	60.4 (34.2, 106.5)	37	35.0 (20.3, 60.4)
Placebo vaccine	69	15.9 (9.9, 25.6)	27	40.1 (24.9, 64.5)	19	84.0 (42.8, 164.8)	12	33.6 (16.9, 67.0)
Active vaccine	65	24.4 (15.2, 39.3)	57	26.4 (17.7, 39.4)	34	44.1 (23.1, 84.0)	25	36.4 (18.7, 70.6)
**Human treatment**	112	8.6 (5.9, 12.4)	91	11.3 (7.6, 16.7)	22	14.3 (7.9, 25.7)	12	17.5 (9.6, 31.8)
Placebo vaccine	44	7.8 (4.8, 12.9)	40	8.5 (5.6, 13.1)	9	8.9 (4.4, 18.1)	5	11.4 (5.3, 24.7)
Active vaccine	68	9.3 (5.8, 15.0)	51	14.8 (9.9, 22.3)	13	22.5 (11.3, 44.7)	7	26.4 (12.5, 55.6)
**Mollusciciding**	131	21.7 (15.0, 31.2)	121	20.9 (14.2, 30.7)	41	13.6 (7.7, 24.2)	22	15.5 (8.8, 27.5)
Placebo vaccine	70	23.5 (14.6, 37.7)	68	28.1 (19.2, 41.2)	28	12.3 (6.4, 23.6)	15	12.3 (6.2, 24.4)
Active vaccine	61	19.9 (12.3, 32.2)	53	15.4 (10.2, 23.2)	13	15.2 (7.6, 30.4)	7	19.9 (9.4, 42.3)
Total	377	15.5 (11.3, 21.2)	296	19.5 (14.0, 27.1)	116	22.9 (13.7, 38.5)	71	21.5 (14.4, 32.1)

* *Control, group received the placebo or SjCTPI vaccine only. Discrepancies occur in the number positive and the number with non-zero GMEPG in Table 2: In 2010 1 person was MHT positive and had a KK egg count of zero; in 2012, 1 person was MHT positive and did not have a KK egg count measured, and 41 persons had a KK egg count of zero; in 2013 these figures were 1 and 17, respectively*.

Infection intensity varied significantly among intervention arms at baseline, with intensities being significantly lower in the human chemotherapy arm compared to the control arm and mollusciciding arm (*P* < 0.001 for both comparisons). Infection intensity was significantly higher in the active vaccine group compared with the placebo group within the control arm (*P* < 0.001). Infection intensity increased over time to 2013, although this was variable and more apparent in the control and human chemotherapy arms.

### Bovines

All bovines (a total of 468 animals) present in the 12 villages were examined at baseline, with a prevalence of infection of 11.8% and an infection intensity of 5.5 GMEPG. Baseline prevalence and infection intensity from 2010 to 2013 are shown by intervention group in Tables 4, 5. There was no significant variation in bovine infection prevalence or intensity at baseline.

**Table 4:** Bovine infection (%) by intervention group and year.

**Group**	**2010**		**2011**		**2012**		**2013**	
	**Number tested**	**Prevalence** **(95% CI)**	**Number tested**	**Prevalence** **(95% CI)**	**Number tested**	**Prevalence** **(95% CI)**	**Number tested**	**Prevalence** **(95% CI)**
^*^Control	145	10.3 (6.3, 16.5)	123	6.5 (3.1, 13.0)	94	6.4 (2.9, 13.5)	64	3.5 (0.3, 30.9)
Placebo vaccine	70	11.4 (5.8, 21.3)	67	6.0 (2.3, 14.9)	40	7.5 (2.4, 20.9)	26	0
Active vaccine	75	9.3 (4.5, 18.3)	56	7.1 (2.7, 17.6)	54	5.6 (1.8, 15.9)	38	8.3 (1.6, 34.07)
**Human treatment**	174	13.2 (8.9, 19.1)	166	10.3 (6.1, 17.0)	158	3.8 (1.7, 8.2)	160	3.6 (0.5, 21.1)
Placebo vaccine	62	14.5 (7.7, 25.6)	57	8.8 (3.7, 19.4)	58	3.4 (0.9, 12.8)	56	7.3 (1.6, 27.9)
Active vaccine	112	12.5 (7.5, 20.0)	109	11.0 (6.3, 18.4)	100	4.0 (1.5, 10.2)	104	1.4 (0.2, 9.6)
**Mollusciciding**	149	11.4 (7.2, 17.6)	118	12.2 (6.9, 20.6)	113	4.4 (1.8, 10.2)	83	21.8 (3.2, 70.5)
Placebo vaccine	92	7.6 (3.7, 15.1)	68	7.4 (3.1, 16.5)	63	3.2 (0.8, 11.9)	53	13.8 (3.1, 44.8)
Active vaccine	57	17.5 (9.7, 29.7)	50	18.0 (9.6, 31.2)	50	6.0 (1.9, 17.1)	30	56.9 (17.3, 89.3)
Total	468	11.8 (9.1, 15.0)	407	9.6 (7.1, 12.8)	365	4.7 (2.9, 7.4)	307	6.6 (1.9, 20.3)

* *Control, group received the placebo or SjCTPI vaccine only*.

**Table 5:** Bovine infection intensity (GMEPG) in infected bovines by intervention group and year.

**Group**	**2010**		**2011**		**2012**		**2013**	
	**Number positive**	**GMEPG** **(95% CI)**	**Number positive**	**GMEPG** **(95% CI)**	**Number positive**	**GMEPG** **(95% CI)**	**Number positive**	**GMEPG** **(95% CI)**
^*^Control	15	5.1 (2.3, 11.1)	8	3.4 (1.0, 11.1)	6	13.2 (5.4, 32.4)	4	2.2 (0.9, 4.6)
Placebo vaccine	8	5.3 (1.8, 15.3)	4	2.8 (0.4, 17.9)	3	8.5 (2.6, 27.4)	0	–
Active vaccine	7	5.0 (1.6, 15.3)	4	3.9 (0.6, 24.9)	3	20.4 (6.3, 65.8)	4	2.1 (0.9, 4.6)
**Human treatment**	23	4.4 (2.7, 8.7)	17	3.4 (1.3, 9.8)	6	4.4 (1.8, 10.9)	6	1.8 (0.9, 3.4)
Placebo vaccine	9	7.7 (2.7, 21.6)	5	1.9 (0.1, 11.7)	2	4.6 (1.1, 19.3)	4	1.3 (0.6, 2.9)
Active vaccine	14	2.9 (1.2, 7.1)	12	5.2 (1.1, 23.9)	4	4.3 (1.6, 11.9)	2	3.1 (1.0, 9.7)
**Mollusciciding**	17	8.9 (4.2, 19.0)	14	1.1 (0.4, 3.1)	5	1.6 (0.6, 4.3)	28	4.9 (3.6, 6.6)
Placebo vaccine	7	8.7 (2.8, 26.8)	5	1.2 (0.2, 6.9)	2	4.1 (1.0, 17.1)	11	4.4 (2.8, 7.1)
Active vaccine	10	9.1 (3.3, 24.7)	9	1.0 (0.2, 5.0)	3	0.9 (0.3, 2.8)	17	5.3 (3.6, 7.8)
Total	55	5.8 (3.8, 8.7)	39	2.3 (1.2, 4.3)	17	4.5 (2.3, 9.1)	38	3.1 (1.8, 5.2)

* *Control, group received the placebo or SjCTPI vaccine only*.

The bovine vaccine coverage was high, ranging from 75.6 to 91.6%, overall 83.9% (Table 6). The vaccine and PZQ treatment of bovines were well-tolerated with no direct adverse effects recorded.

**Table 6:** Bovine vaccine coverage by intervention group.

**Group**	**Number**	**Expected number of doses**	**Number of doses given**	**Coverage %**
^*^Control	131	470	418	88.9
Placebo vaccine	68	233	201	86.3
Active vaccine	63	237	217	91.6
**Human treatment**	155	545	452	82.9
Placebo vaccine	64	225	183	81.3
Active vaccine	91	330	269	81.5
**Mollusciciding**	119	437	356	81.5
Placebo vaccine	63	236	204	86.4
Active vaccine	56	201	152	75.6
Total	405	1,462	1,226	83.9

* *Control, group received the placebo or SjCTPI vaccine only*.

### Modeling of Human Results

Table 7 shows the results of fitting the logistic regression of human incident infection. Averaged over other intervention arms and the post-baseline period, there was no significant difference between active vaccine and placebo vaccine groups, although the active vaccine group had higher rates of infection in 2011 [OR = 1.44 95% CI (1.11, 1.86)]. Overall, human chemotherapy and mollusciciding showed significant protective effects in 2013 [OR = 0.55 (0.31, 0.96), OR = 0.55 (0.32, 0.96), respectively]. The excess of infections within the active vaccine group was specifically marked in the control arm [OR = 2.27 (1.52, 3.39)]. Over the post-baseline period, human treatment and mollusciciding separately showed a halving of infection rates [OR = 0.48 (0.33, 0.70), OR = 0.50 (0.34, 0.76), respectively] within the active vaccine group.

**Table 7:** Odds ratios (95% confidence intervals) and *P*-values for treatment effects on human incident infection, adjusted for baseline infection, sex, and age group; derived from logistic regression using Generalized Estimating Equations (GEEs) for correlated data; participants satisfying initial inclusion criteria, with baseline infection status measured and at least one follow-up stool measurement.

	**2011**		**2012**		**2013**		**All years**	
	**OR (95% CI)**	***P***	**OR (95% CI)**	***P***	**OR (95% CI)**	***P***	**OR (95% CI)**	***P***
**OVERALL EFFECTS**								
Vaccine	1.44 (1.11, 1.86)	0.0066	1.21 (0.85,1.73)	0.29	0.90 (0.57,1.43) 1.43)	0.65	1.16 (0.92,1.47) 1111.47)	0.22
Human treatment	1.19 (0.85, 1.67)	0.31	0.74 (0.47,1.16)	0.19	0.55 (0.31,0.96)	0.035	0.78 (0.59, 1.05)	0.10
Mollusciciding	1.28 (0.94, 1.74)	0.12	0.86 (0.56, 1.30)	0.47	0.55 (0.32,0.96) 0.96)	0.035	0.85 (0.64, 1.13)	0.25
**VACCINE EFFECT**								
Control arm	2.30 (1.39, 3.80)	0.001	2.86 (1.56, 5.24)	<0.001	1.78 (0.87,3.62) 3.62)	0.11	2.27 (1.52, 3.39)	<0.001
Human treatment arm	1.33 (0.84, 2.10)	0.23	0.81 (0.41, 1.58)	0.53	0.59 (0.25,1.40) 1.40)	0.23	0.86 (0.56, 1.32)	0.48
Mollusciciding arm	0.98 (0.69, 1.38)	0.89	0.77 (0.44, 1.37)	0.38	0.69 (0.30, 1.59) 1.1.59)	0.39	0.80 (0.54, 1.20)	0.29
**HUMAN TREATMENT**								
Placebo vaccine	1.57 (0.93, 2.65)	0.093	1.39 (0.70, 2.76)	0.35	0.95 (0.43, 2.13)	0.9	1.28 (0.82, 2.00)	0.29
Active vaccine	0.91 (0.59, 1.40)	0.66	0.39 (0.22, 0.71)	0.002	0.32 (0.15, 0.69)	0.004	0.48 (0.33, 0.70)	<0.001
**MOLLUSCICIDING**								
Placebo vaccine	1.96 (1.22, 3.14)	0.005	1.65 (0.88, 3.09)	0.12	0.89 (0.41, 1.92)	0.77	1.42 (0.95, 2.12)	0.084
Active vaccine	0.83 (0.56, 1.23)	0.36	0.45 (0.26, 0.77)	0.004	0.35 (0.16, 0.75)	0.008	0.50 (0.34, 0.76)	<0.001

Table 8 shows the results of fitting the log-transformed intensity (GMEPG) regression model. The active vaccine had no overall effect on intensity. Within those receiving human treatment, the active vaccine was associated with an increase in intensity. Human treatment and mollusciciding were associated with reductions in GMEPG of ~60%: OR: 0.36 (0.29, 0.45) and 0.38 (0.31, 0.48), respectively.

**Table 8:** Relative increases in human GMEPG (95% confidence intervals) among positives and *P*-values for treatment effects, adjusted for baseline infection, sex, and age group; derived from logistic regression using GEEs for correlated data; participants satisfying initial inclusion criteria, with baseline infection status measured and at least one follow-up stool measurement.

	**2011**		**2012**		**2013**		**All years**	
	**OR (95% CI)**	***P***	**OR (95% CI)**	***P***	**OR (95% CI)**	***P***	**OR (95% CI)**	***P***
**OVERALL EFFECTS**								
Vaccine	0.86 (0.67, 1.09)	0.20	0.97 (0.73, 1.29)	0.86	1.16 (0.76, 1.78)	0.49	0.99 (0.81, 1.21)	0.92
Human treatment	0.35 (0.26, 0.48)	<0.001	0.26 (0.19, 0.35)	<0.001	0.51 (0.31, 0.82)	0.006	0.36 (0.29, 0.45)	<0.001
Mollusciciding	0.60 (0.43, 0.83)	0.002	0.24 (0.17, 0.33)	<0.001	0.39 (0.26, 0.60)	<0.001	0.38 (0.31, 0.48)	<0.001
**VACCINE EFFECT**								
Control arm	0.61 (0.36, 1.05)	0.08	0.51 (0.38, 0.68)	<0.001	1.14 (0.86, 1.51)	0.38	0.71 (0.56, 0.88)	0.002
Human treatment arm	1.83 (1.46, 2.29)	<0.001	2.22 (1.26, 3.91)	0.006	1.59 (0.63, 4.04)	0.33	1.86 (1.24, 2.79)	0.003
Mollusciciding arm	0.56 (0.37, 0.85)	0.007	0.83 (0.46, 1.47)	0.51	0.87 (0.38, 1.98)	0.74	0.74 (0.50, 1.09)	0.13
**HUMAN TREATMENT**								
Placebo vaccine	0.21 (0.13, 0.34)	<0.001	0.12 (0.07, 0.22)	<0.001	0.43 (0.19, 0.97)	0.042	0.22 (0.15, 0.33)	<0.001
Active vaccine	0.61 (0.45, 0.84)	0.002	0.54 (0.40, 0.73)	<0.001	0.60 (0.35, 1.02)	0.06	0.58 (0.46, 0.74)	<0.001
**MOLLUSCICIDING**								
Placebo vaccine	0.63 (0.37, 1.06)	0.08	0.19 (0.13, 0.27)	<0.001	0.45 (0.31, 0.64)	<0.001	0.37 (0.29, 0.48)	<0.001
Active vaccine	0.57 (0.38, 0.87)	0.009	0.30 (0.18, 0.52)	<0.001	0.34 (0.16, 0.75)	0.007	0.39 (0.27, 0.57)	<0.001

### Modeling of Bovine Results

An overall model could not be fitted to bovine infection owing to zero prevalence in the placebo group within the control arm. Separate models at 2011 and 2012 showed no significant variation in infection rates among intervention arms. A model for 2013 excluding the placebo vaccine group within the control arm showed significant variation in the remaining five intervention arm/groups, although there was no consistent pattern with the active vaccine vs. placebo vaccine. The active vaccine group within the human chemotherapy arm had significantly lower infection rates at 2013, compared to average; and both mollusciciding groups had significantly higher rates of infection.

### Bovine Serology

**Figure 2:**
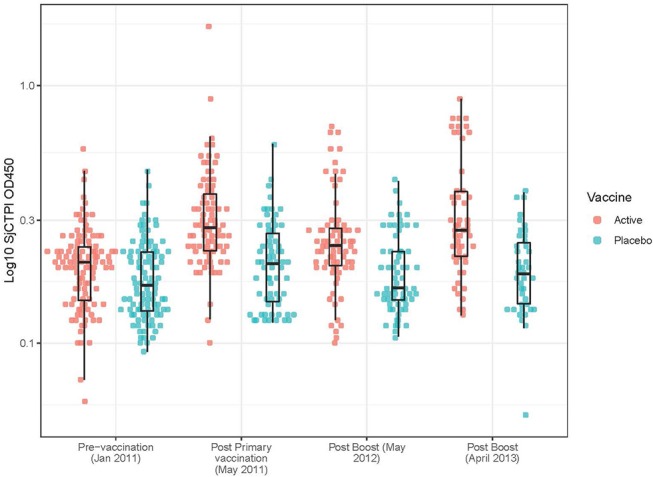
Anti-SjCTPI antibody OD_450_ levels in bovine serum samples for all collection time points. The anti-SjCTPI IgG antibody levels (OD_450_) were measured in sera from individual bovines collected pre-vaccination (January 2011), post primary vaccination (May 2011), post boost (May 2012), and post boost (April 2013). Anti-SjCTPI antibody levels are compared for bovines given active vaccine or placebo for all the collection time points. The box and whisker plot display the median (central horizonal line), first and third quartiles (bottom and top of box, respectively; inter-quartile range), and values within 1.5 times the inter-quartile range of the first and third quartiles (vertical lines).

The active bovine vaccine group had a significantly higher level of anti-SjCTPI IgG antibodies and a significantly lower level of anti-SjC SEA IgG antibodies compared with the placebo vaccinated group after receiving the priming vaccination and primary boost (Table 9; Figure 2; Supplementary Figures 4, 5). Western blot analysis of a subset of individual serum samples obtained from the active priming vaccinated/boosted group at this time point (May 2011) showed the reaction was specific as sera from placebo-vaccinated bovines did not react with SjCTPI (data not shown). The SjCTPI antibody response was maintained after the second boost and increased after the third boost. There was no significant difference in anti-SjC SEA response after either booster (Table 9; Supplementary Figure 5).

**Table 9:** Geometric Mean (GM) antibody response (OD_450_) in bovines given vaccine or placebo, for each blood sample.

**Vaccine**		**Pre-vaccination** **(Jan 2011)**		**Post primary vaccination** **(May 2011)**		**Post boost** **(May 2012)**		**Post boost** **(April 2013)**
	***N***	GM OD^*^ **(95% CI)**	***N***	**GM OD** **(95% CI)**	***N***	**GM OD** **(95% CI)**	***N***	**GM OD** **(95% CI)**
**SjC SEA**								
Placebo vaccine	83	0.266 (0.240, 0.294)	60	0.383 (0.344, 0.425)	53	0.286 (0.261, 0.312)	39	0.296 (0.264, 0.332)
Active vaccine	82	0.274 (0.249, 0.301)	69	0.301 (0.278, 0.326)	64	0.296 (0.272, 0.323)	46	0.293 (0.271, 0.316)
*P* value		0.66		<0.001		0.56		0.86
**SjCTPI**								
Placebo vaccine	83	0.174 (0.162, 0.187)	60	0.210 (0.191, 0.231)	53	0.181 (0.165, 0.200)	39	0.192 (0.169, 0.217)
Active vaccine	82	0.197 (0.182, 0.213)	69	0.294 (0.264, 0.327)	64	0.234 (0.213, 0.258)	46	0.298 (0.256, 0.348)
*P* value		0.34		<0.001		<0.001		<0.001

* *GM OD, Geometric Mean Optical Density*.

The GM OD for anti-SjCTPI antibodies was significantly higher in the active vaccine group than the placebo vaccine at all post-vaccination time points (Table 9; Supplementary Figure 4). The GM OD for anti-SjC SEA antibodies was significantly higher in the placebo vaccine group at the first post-vaccination time point in 2011 only (Table 9; Supplementary Figure 5).

The levels of anti-SjCTPI antibodies were negatively correlated with bovine egg counts within the active vaccine group at all post-vaccination time points, although there was a positive correlation pre-vaccination (Table 10). Anti-SjC SEA antibody levels were positively correlated with bovine egg counts after the second booster injection (2012) within this group. Within the placebo vaccine group, anti-SjCTPI antibody levels were negatively correlated with egg counts after the second booster injection (Table 10).

**Table 10:** Spearman's correlations (r_s_) between bovine antibody responses to vaccine or placebo and bovine egg counts over the trial course.

		**SjC SEA**				**SjCTPI**			
		**Pre-vaccination (Jan 2011)**	**Post primary vaccination (May 2011)**	**Post booster** **(May 2012)**	**Post booster** **(April 2013)**	**Pre-vaccination (Jan 2011)**	**Post primary vaccination (May 2011)**	**Post booster** **(May 2012)**	**Post booster** **(April 2013)**
All	*N*	93	71	82	75	93	71	82	75
	r_s_	−0.112	0.018	0.311	0.214	−0.173	−0.117	−0.329	−0.193
	*P-*value	0.29	0.88	0.004	0.065	0.098	0.33	0.003	0.097
Placebo vaccine	*N*	44	29	37	35	44	29	37	35
	r_s_	−0.157	0.205	0.264	0.180	−0.089	0.355	−0.454	−0.177
	*P-*value	0.31	0.29	0.12	0.30	0.57	0.059	0.005	0.31
Active vaccine	*N*	49	42	45	40	49	42	45	40
	r_s_	−0.113	0.037	0.348	0.249	0.312	−0.408	−0.385	−0.324
	*P-*value	0.44	0.82	0.019	0.12	0.029	0.007	0.009	0.041

### Snail Prevalence and Density of Infected Snails

Snail density was highest in the mollusciciding villages at baseline and decreased markedly over time. The density of infected snails decreased in all intervention groups over time (Table 11).

**Table 11:** Snail density (per m2) and density of infected snails (per 100 m^2^).

**Group**	**No sites 2010**	**Snail density 2010**	**Density infected snails 2010**	**No sites 2011**	**Snail density 2011**	**Density infected snails 2011**	**No sites 2012**	**Snail density 2012**	**Density infected snails 2012**	**No sites 2013**	**Snail density 2013**	**Density infected snails 2013**
^*^Control	14	1.94	3.21	16	1.47	0.47	18	0.82	0	16	0.86	0
Placebo vaccine	5	1.63	5.97	6	1.56	0.14	8	0.68	0	6	0.90	0
Active vaccine	9	2.21	0.75	10	1.42	0.10	10	1.04	0	10	0.80	0
**Human treatment**	10	5.41	1.54	20	3.32	0.40	20	2.59	0.13	12	4.90	0
Placebo vaccine	3	0.15	0.92	12	0.05	0.09	12	0.05	0	4	0.10	0
Active vaccine	7	7.30	1.76	8	6.85	0.74	8	5.33	0.28	8	6.77	0
**Mollusciciding**	30	12.4	0.47	36	8.51	0.19	28	4.62	0	26	3.47	0
Placebo vaccine	11	16.85	1.03	12	11.27	0.39	8	8.19	0	8	4.98	0
Active vaccine	19	9.74	0.14	24	6.82	0.07	20	2.38	0	18	2.46	0
Total	54	9.16	1.22	72	6.05	0.25	66	2.98	0.03	54	2.84	0

* *Control, group received the placebo or SjCTPI vaccine only*.

### Mathematical Modeling of Intervention Arms

**Figure 3:**
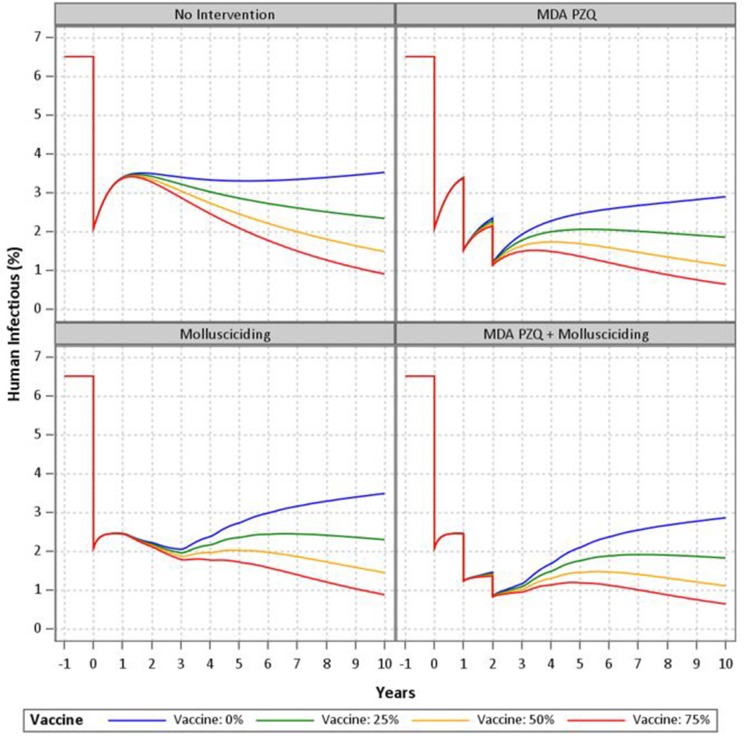
Mathematical modeling of the SjCTPI vaccine and integrated control strategies.

Results of the mathematical modeling are as shown in Figure 3. The non-intervention model with zero vaccine efficacy shows a rebound effect after the initial treatment with steadily increasing prevalence to about half the baseline level after 10 years. Further human treatment results in greater short-term decreases with a similar rebound effect upon cessation. Mollusciciding maintains the post-initial treatment level until its cessation, with subsequent rebound effects and steadily increasing prevalence of infection. The vaccine with 25–75% efficacy has the effect of reducing the rebound after mass drug treatment or mollusciciding and further reducing the longer term prevalence at 10 years to around 1%. The combination of mass drug treatment and mollusciciding halves the effect of the single interventions to about 1% immediately post-trial. This is then maintained and then somewhat further reduced over the 10 year period.

## Discussion

Elimination of schistosomiasis is now on the immediate horizon for P.R. China but it is important to evaluate which intervention combinations will be needed to achieve this goal. We present the outcomes of a double-blind cluster CRT using a multi-factorial design undertaken in Hunan Province in 2010–2014. We evaluated the impact of a combination of human mass chemotherapy, mollusciciding and bovine vaccination—using the SjCTPI vaccine—on the transmission of *S. japonicum*. This is the first reported schistosomiasis field trial of its type and magnitude, and the first to report on the outcomes of a CRT, equivalent to a phase III clinical trial, to test a schistosomiasis transmission blocking vaccine in the field.

Results of the multi-factorial trial revealed that human praziquantel chemotherapy is indeed an effective intervention at the population level showing an efficacy of ~50% on human infection and reinfection. Of particular note was our finding that mollusciciding had an indirect ~50% efficacy on human infection rates; as far as we are aware, this is the first time that such an outcome has been demonstrated.

Serology showed that the SjCTPI vaccine was effective in inducing an antibody response in the bovine cohort. This response was maintained over the course of the trial, and a negative correlation with bovine egg counts was observed at all post-vaccination time points. This is in line with the experimental results obtained with the SjCTPI vaccine (18, 23) and reinforces its potential for inducing specific anti-SjCTPI antibodies in bovine hosts within their natural setting.

**Figure 4:**
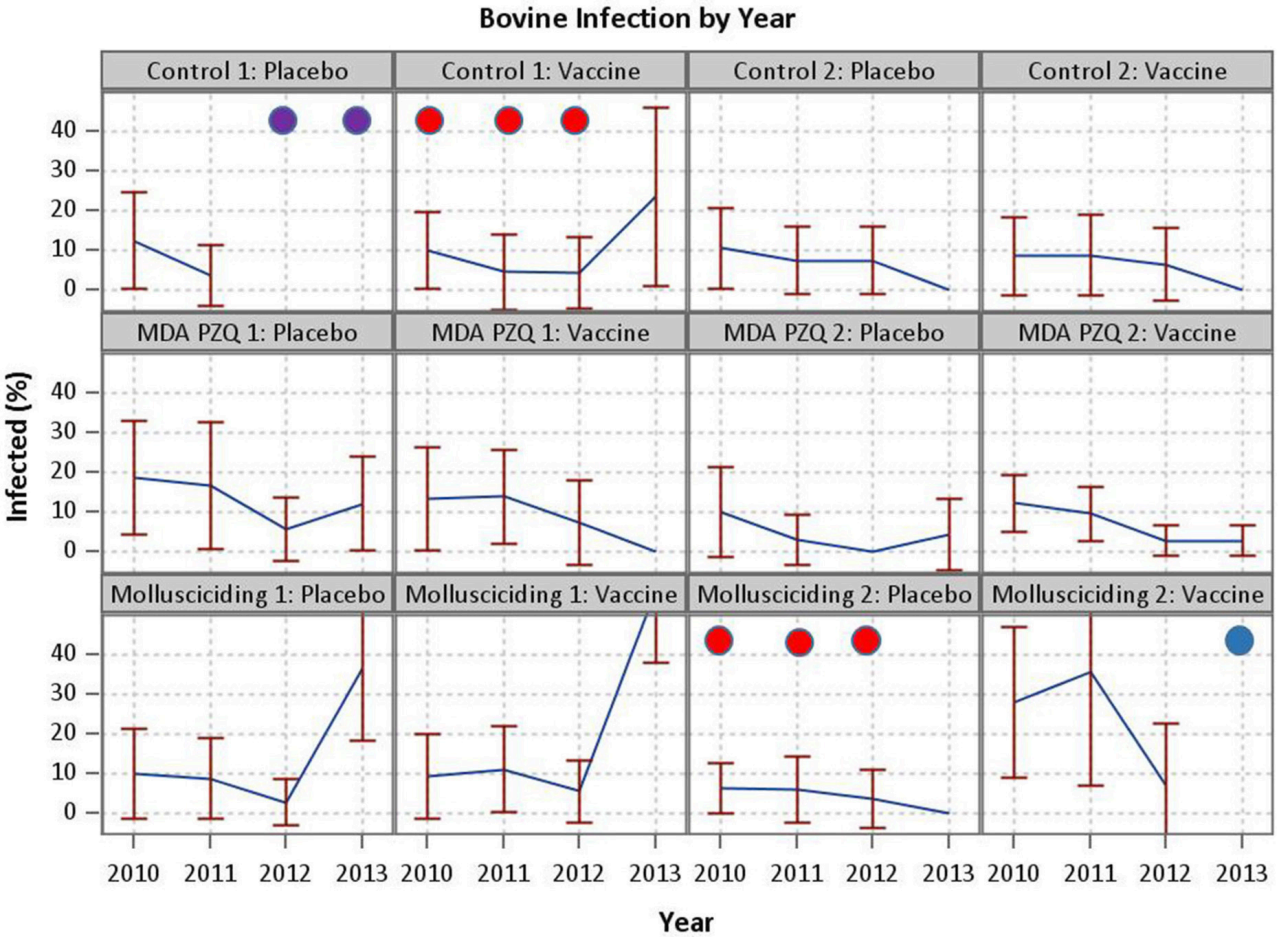
Events compromising the trial. In one control arm/active vaccine village and one mollusciciding arm/placebo vaccine village, infected humans and bovines were PZQ drug-treated from 2010 to 2012 (demarcated by red circles). In one control arm/placebo vaccine village all bovines were slaughtered in 2012 and 2013 to control an outbreak of brucellosis (demarcated by purple circles).In one mollusciciding arm/active vaccine village, the bovines were removed in 2013 (demarcated by a blue circle).

Despite this encouraging outcome, the effect of the SjCTPI vaccine in preventing human infection was inconclusive in that we were unable to show a difference in human infection rates between the active vaccine and placebo vaccine groups. This is likely due to a number of factors over the 4-year trial duration, including: (a). In one control arm/active vaccine village and one mollusciciding arm/placebo vaccine village, infected humans and bovines were PZQ drug-treated from 2010 to 2012; (b). In one control arm/placebo vaccine village all bovines were slaughtered in 2012 and 2013 to control an outbreak of brucellosis; and (c). In one mollusciciding arm/active vaccine village, the bovines were removed in 2013 (Figure 4). These activities were undertaken by the China National Schistosomiasis Control Program over which we had no jurisdiction. In particular, the loss of bovines compromised the trial design, resulting in reduced power and the contamination of outcome measures necessary for comparing the effect of the active vaccine with the placebo vaccine. This highlights the difficulties in undertaking field trials of this nature and magnitude, particularly over a long period. Mathematical modeling results (discussed below) are thus important in predicting the potential impact of the intervention measures employed.

Mathematical modeling has been used to compare and evaluate the impact of various strategies implemented for the control and elimination of schistosomiasis in China (17, 24–28). Here we used an updated version of the Williams *et al* model (17) to simulate the trial and the combination of interventions employed (human mass chemotherapy, snail control through mollusciciding, and bovine vaccination). The modeling clearly demonstrates that one intervention alone will not work to eliminate schistosomiasis and indicates that an approach integrating multiple interventions would be the most effective in bringing down transmission and sustaining the impact of a control program. Depending on the level of immunological protection, the vaccine was shown to be effective in reducing the rebound in human infection to varying degrees following human treatment and snail control through mollusciciding. A 75% efficacious vaccine in combination with these two other interventions was shown to result in *S. japonicum* elimination (i.e., <1% prevalence).

The Chinese Government continues in its commitment toward the control of schistosomiasis, developing the new national elimination plan for the period 2016–2020 (10, 29). MDA is now used biannually in the lake areas among boat people and fisher communities living close to water-bodies infested with infected oncomelanid snails. Other high-risk populations with extensive water contact are subjected to questionnaire surveys or serology prior to selective PZQ treatment. Extensive drug treatment of bovines has not reduced schistosomiasis prevalence to acceptable levels and in, some areas, the replacement of these animals with motorized tractors has proved more effective in reducing transmission. Any control program with the goal of schistosomiasis elimination must be mindful of long-term sustainability. Much of China's success in its goal of achieving the elimination of schistosomiasis can be attributed to strong, long-term government commitment and support. The Chinese have recognized that a comprehensive multi-facetted approach is most effective, but that any introduced interventions need to be adapted to local conditions and the associated economic costs need careful consideration. Further, it is clear from the experiences, not only in China but also the Philippines, Cambodia, and the Lao People's Democratic Republic that preventive chemotherapy as the sole intervention is not sufficient to interrupt transmission of Asian schistosomiasis (30).

However, for the long-term sustainability and effectiveness of elimination efforts, preventive measures will become increasingly important. The management of feces of fishermen in areas with persistent transmission and further reducing the infection prevalence in livestock are challenges that need addressing (30). A transmission blocking vaccine targeting bovines for the prevention of *S. japonicum* with the required protective efficacy would be invaluable along with other preventive intervention measures such as health education and environmental modification for snail control in China. Schistosomiasis vaccine development has, however, proven highly challenging and it is a stark reality that no vaccine with a sufficient level of protective efficacy is currently available for schistosomiasis. Nevertheless, it is likely that the inclusion of effective anti-schistosome vaccines as components of an integrated intervention package will be required if long term and universal control efforts against this disease are to prove successful. Consequently, funding for vaccine research and the development of more specific, sensitive, rapid and cost-effective diagnostic, and snail survey tools for active surveillance should be strongly advocated if the goal of eliminating schistosomiasis from China, and elsewhere, is to become a reality (31–33).

## Data Availability

The datasets generated for this study are available on request to the first author, Professor Gail Williams.

## Ethics Statement

The CRT was approved by the Animal Ethics and Human Research Ethics Committees of Queensland Institute of Medical Research and the Ethical Committee of Hunan Institute of Parasitic Diseases. Written informed consent was obtained from all adults and from parents or guardians of minors who were involved in the project. Study participants identified as stool egg-positive for schistosomiasis were treated with praziquantel at the recommended dosage (40 mg/kg body weight) of the World Health Organization (34). The trial was registered with the Australia and New Zealand Clinical Trial Registry (ACTRN12611000193976).

## Author Contributions

GW, Y-SL, DG, AR, and DM designed the study. GW, Y-SL, DG, BG, and DM undertook the trial data management and analysis. DH advised on the vaccine regimen and, with LS, provided the active and placebo vaccines. PD, MH, and HY undertook the serology and serological analysis. Y-SL, Z-YZ, S-ML, XY, ZF, J-GG, JZ, Y-LD, and YL undertook all data entry and/or field work for the trial. DM, GW, and DG drafted the manuscript. All authors read and commented on the final version of the paper prior to submission.

### Conflict of Interest Statement

The authors declare that the research was conducted in the absence of any commercial or financial relationships that could be construed as a potential conflict of interest.
