# Supplementary material for: Field Testing Integrated Interventions for Schistosomiasis Elimination in the People's Republic of China: Outcomes of a Multifactorial Cluster-Randomized Controlled Trial
Source: Front Immunol. 2019 Apr 3;10:645. doi: 10.3389/fimmu.2019.00645 (PMC6456715; doi:10.3389/fimmu.2019.00645)
Supplement: Supplementary file 1 [file Data_Sheet_1.pdf]

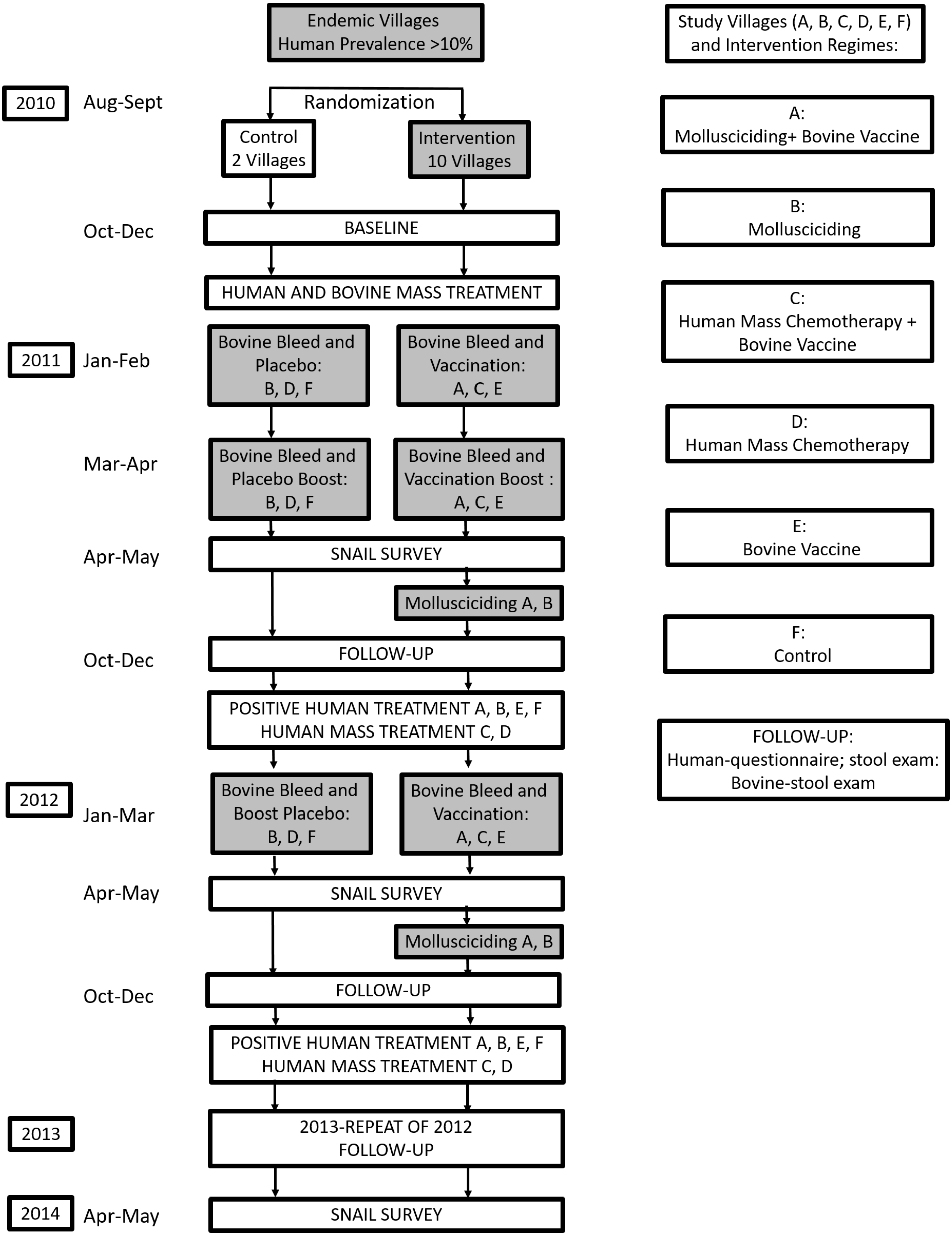

Supplementary Figure 1. Trial profile. Reprinted from Gray et al. (ref. 19) with permission from Elsevier.
