# Supplementary material for: Field Testing Integrated Interventions for Schistosomiasis Elimination in the People's Republic of China: Outcomes of a Multifactorial Cluster-Randomized Controlled Trial
Source: Front Immunol. 2019 Apr 3;10:645. doi: 10.3389/fimmu.2019.00645 (PMC6456715; doi:10.3389/fimmu.2019.00645)
Supplement: Supplementary file 3 [file Data_Sheet_3.PDF]

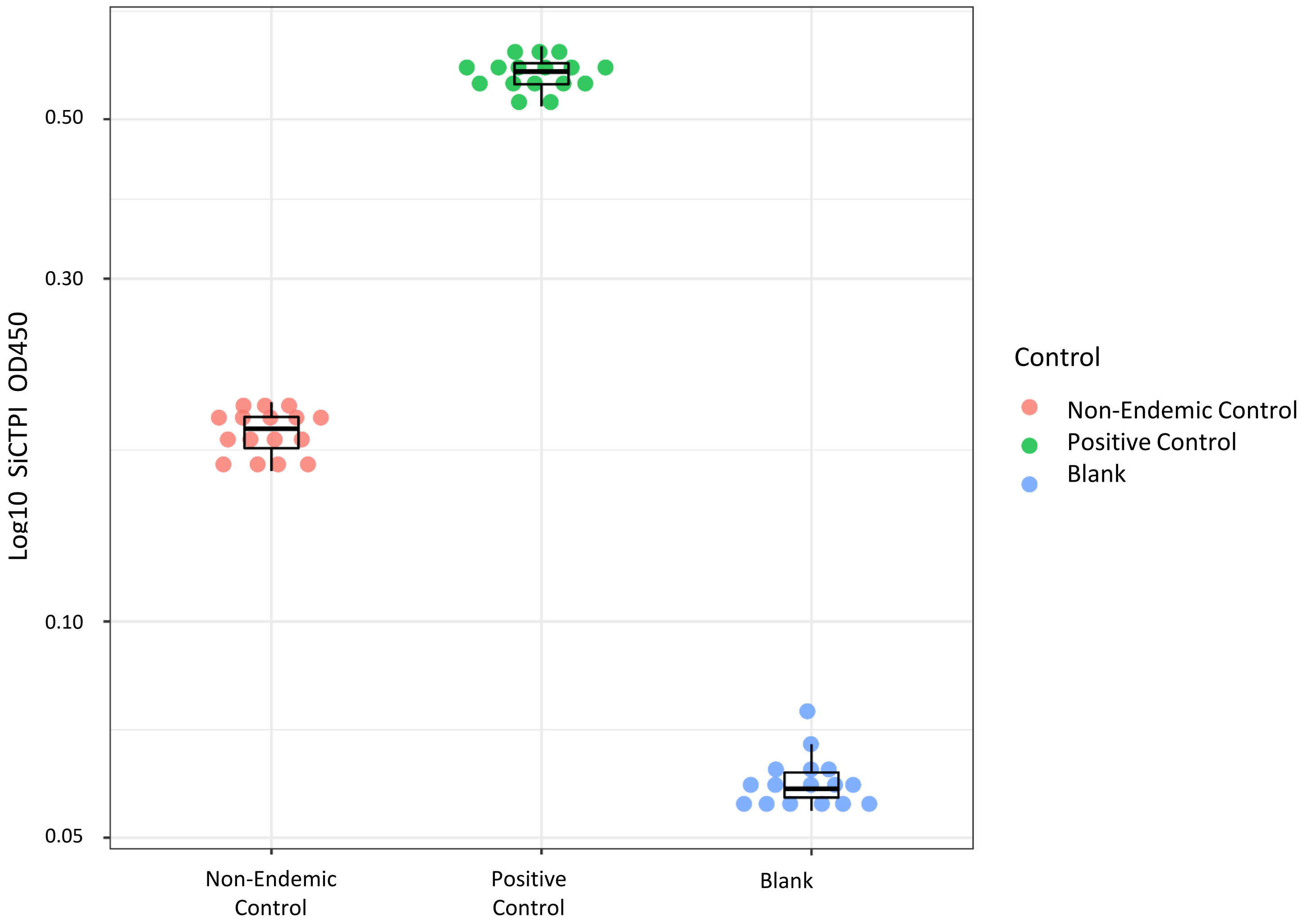

Supplementary Figure 3: Anti-SjC SEA antibody OD_{450} levels in control bovine serum samples. The anti-SjC SEA IgG antibody levels (OD_{450}) were measured in highly reactive sera collected from bovines in schistosomiasis endemic areas (Positive Controls), sera collected from bovines in non-endemic areas (Non-Endemic Controls) and blank wells by indirect ELISA. The box and whisker plot display the median (central horizonal line), first and third quartiles (bottom and top of box, respectively; inter-quartile range), and values within 1.5 times the inter-quartile range of the first and third quartiles (vertical lines).
