# Supplementary material for: Field Testing Integrated Interventions for Schistosomiasis Elimination in the People's Republic of China: Outcomes of a Multifactorial Cluster-Randomized Controlled Trial
Source: Front Immunol. 2019 Apr 3;10:645. doi: 10.3389/fimmu.2019.00645 (PMC6456715; doi:10.3389/fimmu.2019.00645)
Supplement: Supplementary file 4 [file Data_Sheet_4.PDF]

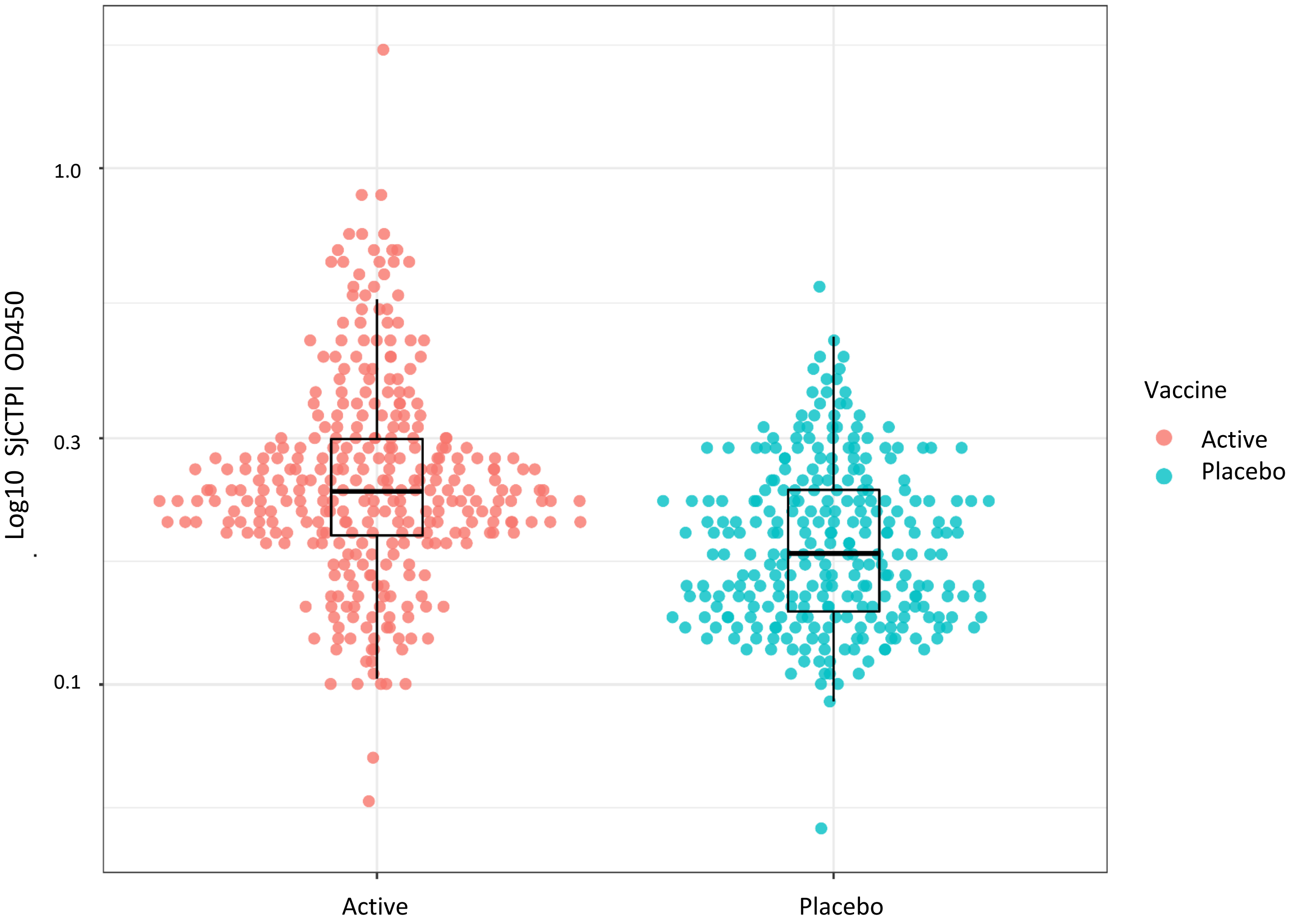

Supplementary Figure 4: Anti-SjCTPI antibody OD_{450} levels in bovine serum samples for all post-vaccination collection times. The anti-SjCTPI IgG antibody levels (OD_{450}) were measured in sera from individual bovines collected post primary vaccination (May 2011), post boost (May 2012) and post boost (April 2013) by indirect ELISA. Anti-SjCTPI antibody levels are compared for all bovines given active vaccine or placebo. The box and whisker plot display the median (central horizonal line), first and third quartiles (bottom and top of box, respectively; inter-quartile range), and values within 1.5 times the inter-quartile range of the first and third quartiles (vertical lines).
