# Supplementary material for: Field Testing Integrated Interventions for Schistosomiasis Elimination in the People's Republic of China: Outcomes of a Multifactorial Cluster-Randomized Controlled Trial
Source: Front Immunol. 2019 Apr 3;10:645. doi: 10.3389/fimmu.2019.00645 (PMC6456715; doi:10.3389/fimmu.2019.00645)
Supplement: Supplementary file 5 [file Data_Sheet_5.PDF]

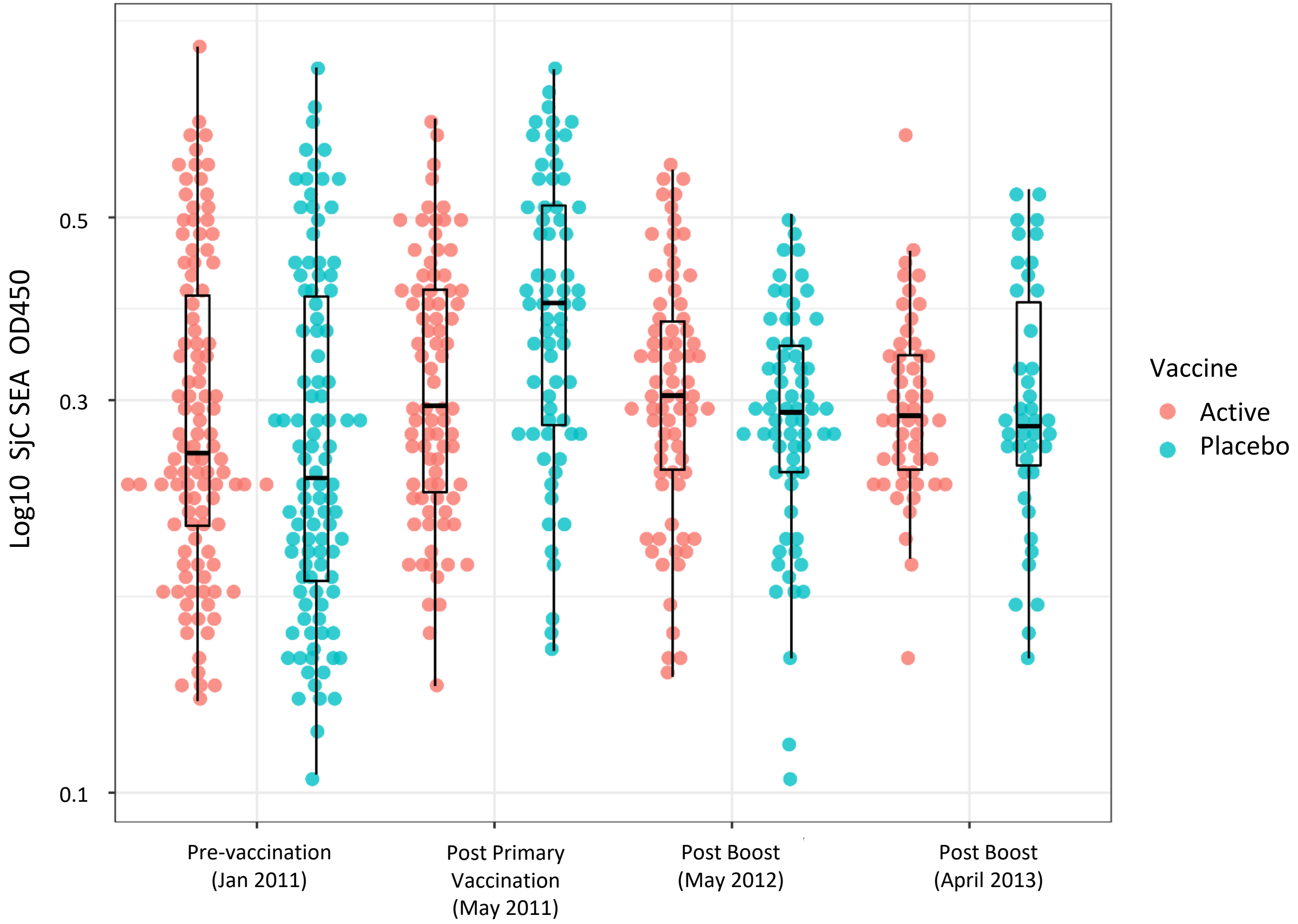

Supplementary Figure 5: Anti-SjC SEA antibody OD_{450} levels in bovine serum samples for all collection time points. The anti-SjC SEA IgG antibody levels (OD_{450}) were measured in sera from individual bovines collected pre-vaccination (Jan 2011), post primary vaccination (May 2011), post boost (May 2012) and post boost (April 2013) by indirect ELISA. Anti-SjC SEA antibody levels are compared for bovines given active vaccine or placebo. The box and whisker plot show the median (central vertical line), first and third quartiles (right and left edge of box, respectively; inter-quartile range), and values within 1.5 times the inter-quartile range of the first and third quartiles (horizontal lines).
